# *Astragalus membranaceus* (Fisch.) Bunge repairs intestinal mucosal injury induced by LPS in mice

**DOI:** 10.1186/s12906-018-2298-2

**Published:** 2018-08-03

**Authors:** Yizhe Cui, Qiuju Wang, Rui Sun, Li Guo, Mengzhu Wang, Junfeng Jia, Chuang Xu, Rui Wu

**Affiliations:** College of Animal Science and Veterinary Medicine, Heilongjiang Bayi Agricultural University, 2# Xinyang Road, New Development District, Daqing, 163319 Heilongjiang China

**Keywords:** *Astragalus membranaceus* (Fisch.) Bunge, Decoction, Mice, Lipopolysaccharide

## Abstract

**Background:**

*Astragalus membranaceus (Fisch.) Bunge* is one of the most widely used traditional Chinese herbal medicines. It is used as immune stimulant, tonic, antioxidant, hepatoprotectant, diuretic, antidiabetic, anticancer, and expectorant. The purpose of the study was to investigate the curative effects of the decoction obtained from *Astragalus membranaceus* root in intestinal mucosal injury induced by LPS in mice. An LPS-induced intestinal mucosal injury mice model was applied in the study.

**Methods:**

The mice were post-treated with *Astragalus membranaceus* decoction (AMD) for 4 days after 3 days LPS induction. ELISA kit was used to detect the content of tumor necrosis factor (TNF)-α, interleukin (IL)-1β, IL-4,IL-6 and IL-8 in the serum of each group mice. The morphological changes in intestinal mucosa at the end of the experiments were observed. Both VH (villus height) and CD (crypt depth) were measured using H&E-stained sections.

**Results:**

There were significant differences in IL-1β, IL-4,IL-6, IL-8 and TNF-α levels in AMD-treated group on the 7th day compared to the controls group. The VH was lower in duodenum, jejunum and the ileum in LPS-treated mice compared to the control animals. Similarly, there was also decrease in V/C. Compared to the control mice, for AMD-treated mice, VH and CD had no significantly differences.

**Conclusions:**

*Astragalus membranaceus* reduced intestinal mucosal damage and promoted tissue repair by inhibiting the expression of inflammatory cytokine.

## Background

Intestinal mucosa is a natural barrier against bacteria. It prevents viruses and other harmful bacteria from entering the blood [1]. Endotoxin is the lipopolysaccharide (LPS) in the cell wall of Gram-negative bacteria, which has a variety of biological activity and is decomposed and released in the process of bacterial metabolism or after death. LPS can stimulate the release of inflammatory mediators from macrophages and neutrophils and eventually lead to the imbalance of inflammatory and anti-inflammatory reactions and the occurrence of excessive systemic inflammation [2].

*Astragalus membranaceus* (Fisch.) Bunge (syn. *Astragalus propinquus* Schischkin) (AM), also known as Huangqi or milk vetch root in China, is an important medicine in traditional Chinese medicine. [3]. This herb possesses many common pharmacological activities, such as multiple organ protection [4, 5], antioxidant [6], hypoglycemic [7], antiviral [8] and so on, and has their own pharmacological properties and mechanisms. Studies have shown that *A. membranaceus* can enhance the contraction of the right ventricular myocardium in rats in a dose-dependent manner [9] and has recently been reported to be a potential promote tissue wound repair. The water extract of *A. membranaceus* is one of the main active preparations obtained from the root of this specie. However, there are not so many reports studies focusing on the decoction of AM. Some studies showed that gastric mucosa and atrophic pathological damage significantly reduced in rats after Huangqi intervention [10]. However, it is still not elucidated whether oral administration of *Astragalus membranaceus* decoction (AMD) could provide a repair effect during intestinal mucosal injury and what is the underlying mechanism. In this study, we explored the repair effect of AMD in LPS induced experimental intestinal mucosal injury in mice.

## Methods

### Drugs and reagents

LPS (*Escherichia coli* O55:B5) and all other chemicals were obtained from Sigma-Aldrich (St. Louis, MO, USA). Distilled water was filtered through a Milli-Q system from EMD Millipore Corporation (Billerica, MA, USA). LPS was suspended in physiological saline and stored as a 20 mg/ml stock. Dilutions prior to injection were into physiological saline. Animals were weighed prior to injection of LPS and stock LPS was diluted to the appropriate dose for each animal.

### Plant material

*Astragalus membranaceus* was purchased from Fu Rui Bang Chinese Medicine Co., Ltd. (Daqing, China), then it was authenticated by Dr. Pengyu Jia and also deposited in Veterinary drug research and Development Center, Heilongjiang Bayi Agricultural University, Heilongjiang, China) according to Chinese Pharmacopoeia (The Pharmacopoeia Commission of PRC, 2010).

### Animals

Male ICR mice weighing 22–25 g were purchased from the Animal Experiment Center of HARBIN MEDICAL UNIVERSITY (DAQING) [Certification no. SYXK (HEI) 2,014,005]. Mice were maintained on a standard light/dark cycle under controlled temperature (22 ± 2 °C) and humidity (50 ± 10%) with certified standard diet and water adlibitum. Mice were habituated to animal facilities for 1 week before the experiment. All the experimental procedures were approved by, and conducted in accordance with Principles of Laboratory Animal Care and according to the rules and ethics set forth by the Ethical Committee of Heilongjiang Bayi Agricultural University.

### Extraction procedure

The general preparation procedure of *Astragalus membranaceus* decoction (AMD) is as follows [11]. Briefly, 100 g the root of *Astragalus membranaceus* was extracted by refluxing with water (1:8, *w*/*v*) for 1.5 h following sonicating for 30 min, then the extraction solutions were combined to be filtered and concentrated to 100 mL under reduced pressure. The concentrations of the residues were 1 g/mL for *Astragalus membranaceus*. Finally, the concentration be adjusted to the required with distilled water for intragastrical administration. After being autoclaved at 100 °C for 20 min, the stock solution was stored at 4 °C.

### Grouping and treatment

**Fig. 1:**
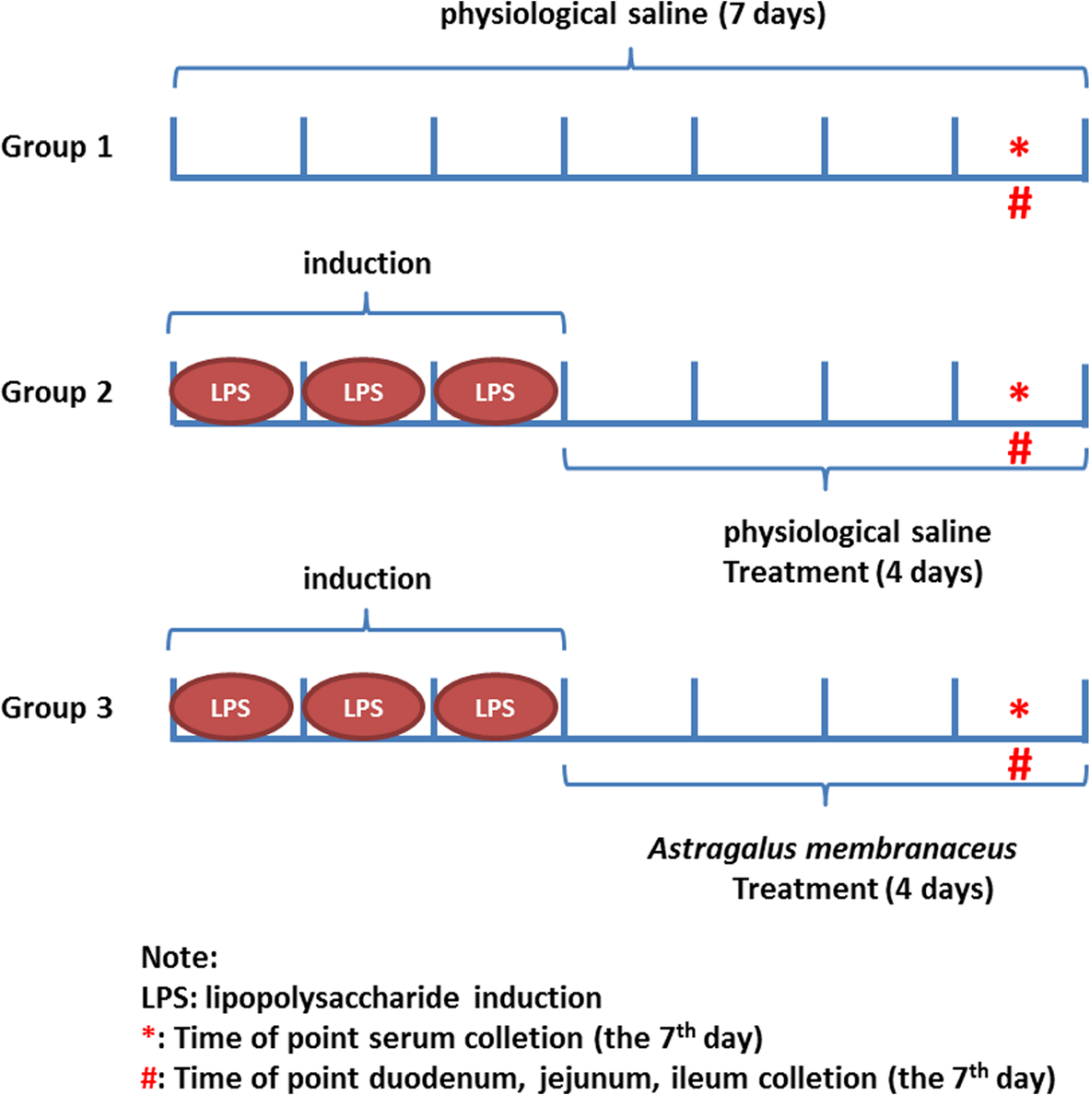
Experimental design and sampling schedule

In experiments, animals were randomized into three groups of ten individuals (Fig. 1). The control group, LPS-treated group and AMD-treated group. Mice in the LPS groups and the AMD group, were intraperitoneally injected with LPS (*Escherichia coli* 055:B5, 5 mg/kg; Sigma) for 3 days. The chosen dose of LPS was based on Die Dai’s study and preliminary experiments [12]. AMD-treated groups were given *Astragalus membranaceus* decoction by intragastric administration once daily and treatments lasted for 4 days after 3 days LPS induction. Briefly, 1 ml syringe with No. 12 gavage needle was used in intragastric administration. The volume of gavage was usually 0.1 ml/10 g body weight. Mice in control group were received physiological saline for 7 days. After euthanizing the mice by carbon dioxide, blood was obtained by cardiac puncture on the 7th day. On collection, blood samples were centrifuged at 5000 rpm for 10 min, and were subsequently stored at − 80 °C before metabolomics analysis. Survivals were recorded for 72 h.

### Determination of inflammatory cytokine levels

Cytokine levels in serum were determined by ELISA by using commercially available kits (Endogen, Cambridge,MA). For each assay, serum was serially diluted to ensure that values obtained were within the linear range of the standards provided with each kit. Each sample was done in duplicate, and data from individual mice were averaged.

### Histopathology

Specimens of the intestinal wall of the duodenum, jejunum and ileum were prepared for histological examination by fixing in 4% formaldehyde-buffered solution, embedding in paraffin, and sectioning. Paraffin sections were cut into slices of 4 μm and stained with H&E staining solution. Finally, the stained sections were observed and photographed under a light microscope (with 100× magnification). Villous height and the associated crypt depth were evaluated using the Image Pro plus 4 analysis software (Media Cybernetics, Baltimore, MD, USA) processing and analysis system. For each intestinal sample, at least 10 well-oriented were measured and the mean value was calculated. The method was the same as described by Nabuurs et al. [13].

### Data analysis

Data were presented as mean and standard deviation (SD). One-way ANOVA showed significant differences among groups. A level of *P* < 0.05 was considered statistically significant. Analysis was performed with the software SPSS version 16.0 (SPSS Inc., USA).

## Results

### Serum concentrations of cytokine

The serum levels of IL-1β, IL-4,IL-6, IL-8 and TNF-α are important biochemical markers for evaluating intestinal mucosal structure and function [14]. In this experiment, the induction of LSP caused significantly higher levels (*P* < 0.05) of IL-1β, IL-4,IL-6, IL-8 and TNF-α in model group on the 7th day compared to the control group (Table 1). Compared with LPS group, the level of inflammatory cytokines decreased significantly (*P* < 0.05) in AMD group. Meanwhile, there were no significant differences of IL-1β, IL-4,IL-6, IL-8 and TNF-α levels in AMD-treated group on the 7th day compared to control group, though the level of IL-4 and IL-1β was higher in AMD group than that in control group, there was no significant differences. The results suggested that AMD had no effect on the immunity of the body, moreover curative treated AMD was effective in ameliorating LPS-induced intestinal mucosal damage.

**Table 1:** Serum levels of cytokines in LPS- and AMD-treated mice

Parameters	Controls	LPS	AMD
TNF-α (pg/mL)	15.64 ± 1.04	50.30 ± 8.26^*^	7.29 ± 1.12
IL-1β (pg/mL)	6.21 ± 0.45	9.36 ± 0.71^*^	7.26 ± 0.45
IL-4 (pg/mL)	3.47 ± 0.33	11.81 ± 0.39^*^	3.65 ± 0.43
IL-6 (pg/mL)	11.34 ± 0.21	14.25 ± 0.36^*^	8.96 ± 0.63
IL-8 (pg/mL)	9.51 ± 1.07	11.86 ± 0.66^*^	7.93 ± 1.13

The data are expressed as the mean ± SD (*n* = 10 per treatment group). ^*^Statistically different from the control group;^*^P < 0.05. Tumor necrosis factor (TNF)-α, interleukin (IL) IL-1β, IL-4,IL-6 and IL-8

### Histopathological changes in intestinal tissue

**Fig. 2:**
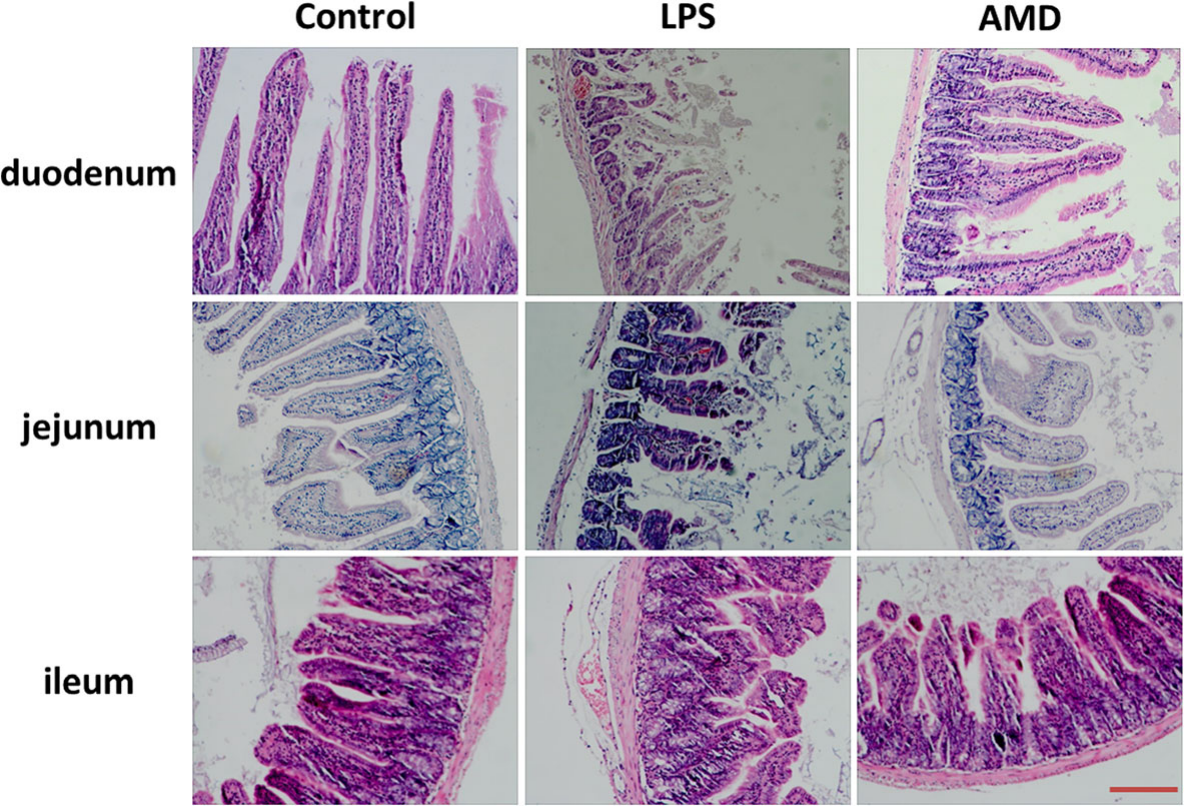
Histomorphometric analyses of intestinal mucosa time changes. Histological appearance of mice intestinal mucosa after haematoxylin and eosin (H&E) stain (original magnification 100×). Scale bars: 50 μm

Pathological examinations of the intestinal mucosal injury were carried out and the LPS-treatment and AMD-treatment are shown in Fig. 2. Compared with the control animals, the pathological changes were obvious, LPS-treated groups caused significant mucosal damage, that is, epithelial shedding, villi fracturing, mucosal atrophy, edema and the villus had shortened on the 7th day after LPS injection (Fig. 2). However, as time goes on, the intestinal mucosa damage begins to recover slowly in the AMD-treated groups on the 7th day. These observations showed that AMD has obvious beneficial effects against intestinal mucosal damage.

### Histomorphological analyses

**Fig. 3:**
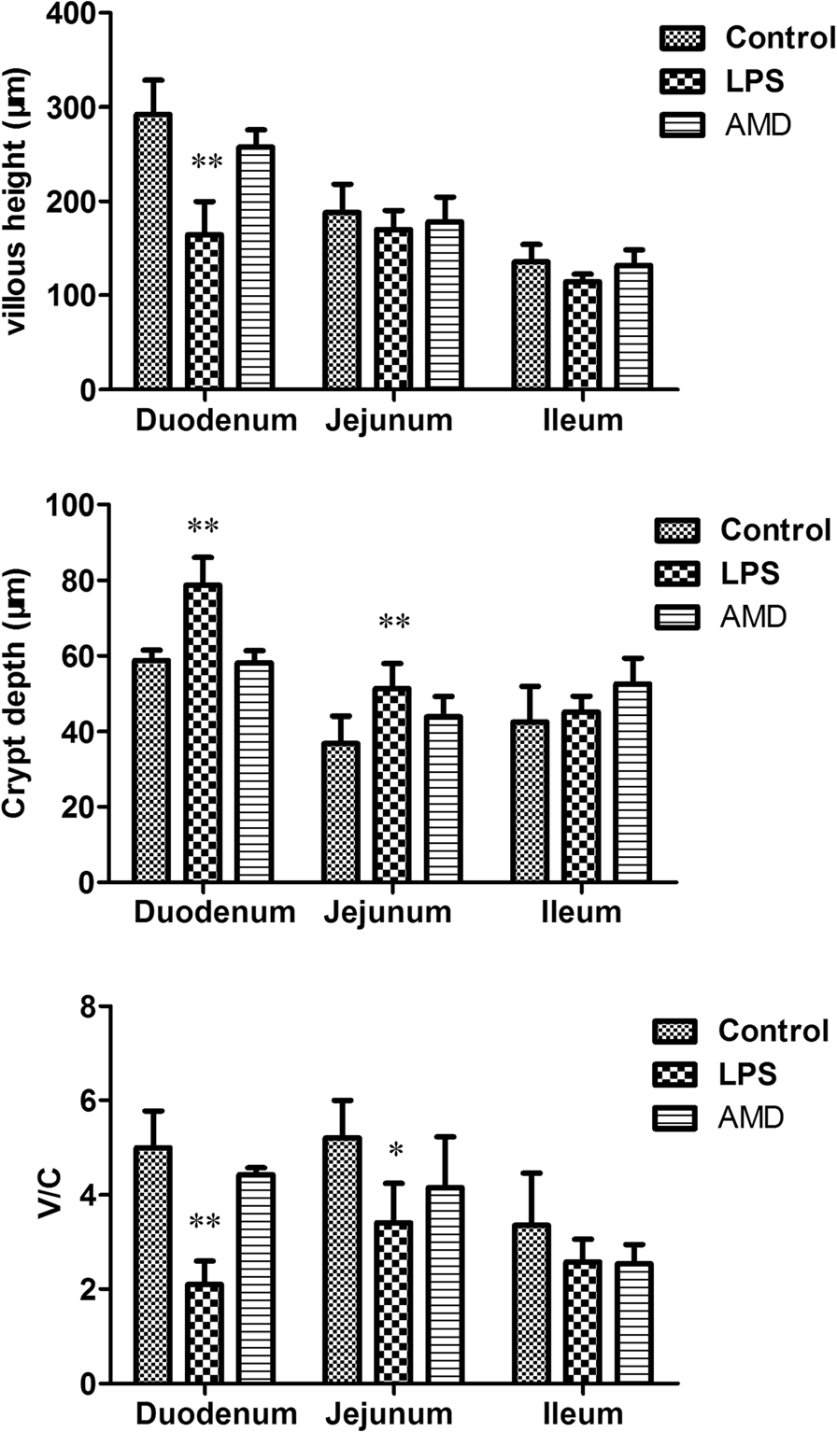
Effects of AMD on VH (villus height), CD (crypt depth) and V/C (villus height /crypt depth), in the duodenum, jejunum and ileum of mice. The data are expressed as the mean ± SD (*n* = 10 per treatment group). Values are significantly different from controls (* *P* < 0.05, ** *P* < 0.01)

The VH and CD, which indicated intestinal villus’s absorptive functions, were measured. The experiments showed that the VH was lower in duodenum, jejunum and the ileum in LPS-treated mice compared to the control animals. Similarly, there was decrease in V/C. Compared to the control mice, for AMD-treated mice, VH and CD had no significantly differences (Fig. 3).

## Discussion

Intestinal mucosal injury is associated with intestinal inflammation [15]. We investigated whether AMD could ameliorate the inflammatory response in mice induced by LPS. A large number of studies suggest that the intestinal ischemia/reperfusion injury, LPS challenge, and intestinal inflammatory diseases can induce the expression of inflammatory cytokines in humans and animals [16]. Both in vitro and in vivo studies show that over-secretory of inflammatory cytokines can have a negative effects on intestinal mucosal integrity, permeability and epithelial function of the intestinal mucosa [17]. The imbalance of cytokine and chemokine secretion plays an important role in mucosal defense. IL-8 is produced by macrophages and epithelial cells. It can chemotaxis and activate neutrophils, which leads to mucous edema, leukocyte infiltration, increased vascular damage and permeability, resulting in immune inflammatory lesions [18]. IL-4 can play a role in pro-inflammatory factors alone in the gut of mice, which can trigger inflammation [19]. The study showed that LPS was identified by Toll like receptor 4 (TLR4) to release TNF-α, IL-1 beta and IL-6 and other cytokines, which mediate and promote the occurrence of inflammatory bowel disease (IBD) [20]. Intraperitoneal injection of LPS can cause intestinal mucosal inflammation, which is characterized by increased inflammatory and anti-inflammatory cytokines. TNF-α plays a major role in causing intestinal inflammation, and its role is to accumulate inflammatory cells to the local tissues of the inflammation, cause edema, activate coagulation cascade, and form granuloma [21]. The common way to treat IBD in clinic is to inhibit TNF-α by using TNF-α antagonist to improve and alleviate IBD symptoms. In this experiment, the mice were intraperitoneally injected with LPS to establish a model of intestinal injury in mice. LPS challenge increased the level of TNF-α, IL-1β, IL-4,IL-6 and IL-8 in the serum (Table 1). Importantly, AMD reduced the concentrations of TNF-α, IL-1β, IL-4,IL-6 and IL-8 in the serum, compared to LPS-challenged mice. These findings indicate that the AMD has beneficial effects in reducing intestinal mucosal inflammation. AMD may inhibit intestinal immune damage, reduce intestinal mucosal edema and promote intestinal mucosal repair by downregulating the expression of cytokine.

The structural characteristics of the small intestinal mucosa are circular folds, intestinal villi and microvilli. These characteristics greatly expand the surface area of the small intestine and make the nutrients fully digested and absorbed in the small intestine. The complete structure of the small intestine is the physiological basis of its digestion and absorption function, and its morphological and structural changes directly affect the surface area of villi, thereby affecting the body’s ability to absorb nutrients [22]. The integrity and height of the intestinal villi determine the absorption area of the small intestine, the absorption of nutrients and the growth of the animals [23]. Therefore, the increase of the villi height, the ratio of the villi/crypt or the decrease of the depth of the recess is related to the improvement of the digestion and absorption of nutrients [24]. Compared with the LPS group, AMD increased the villus height and villus/crypt ratio of the duodenum, as well as the villus height and chorionic ratio of the jejunum and ileum. Crypt depth was significantly reduced in the duodenum and the jejunum, compared with the LPS group. The expression of inflammatory cytokines was consistent with the alteration in the structure of intestinal villi (Table 1). Based on these results, we concluded that AMD protected the intestinal mucosa from the LPS-induced injury.

AM is a well-known medicinal herb for reinforcing Qi (the vital energy) in traditional Chinese medicine [25]. *Astragalus* polysaccharides has the characteristics of antioxidation [26], immunomodulation [27], antiviral, antitumor activities [28] and cardiovascular protection [29]. AM and its active components have been proved to be effective in the treatment of a variety of diseases, such as diabetes mellitus [30] and cardiovascular disorders [31]. In recent years, astragal’s polysaccharides effectively reduced the mucosal damage of experimental colitis in mice by shortening colonic length, reducing colon weight index, and reducing macroscopically and histological scores [32], which is similar to the results of this experiment.

## Conclusions

*Astragalus membranaceus* treatment can protect small intestinal mucosa against LPS injury. Also, *A. membranaceus* promotes tissue repair by inhibiting the expression of inflammatory cytokine. These findings indicate that *A. membranaceus* can partly reduce small intestinal mucosa injury induced by LPS. Further studies of *A. membranaceus* are necessary to develop a new effective plant-derived therapeutic modality for intestinal mucosal injury.

## Abbreviations

AM: *Astragalus membranaceus* (Fisch.) Bunge (syn. *Astragalus propinquus* Schischkin)
AMD: *Astragalus membranaceus* decoction
CD: Crypt depth
H&E: Haematoxylin and eosin
IBD: Inflammatory bowel disease
IL: Interleukin
LPS: Lipopolysaccharide
SD: Standard deviation
TNF: Tumor necrosis factor
V/C: Villus height /Crypt depth
VH: Villus height
